# The Right of the State to Regulate Propagation

**Published:** 1912-08

**Authors:** 


### The Right of the State to Regulate Propagation.

To quote Lord Beaconsfield: "The public health is the foundation on which repose the happiness of the people and the power of a country. The care of the public health is the first duty of a statesman." Most people are ready to concede to the State the right to pass laws regulating the health of the community in a general way, but only a few that would sanction State regulation of matters heretofore considered purely personal. Yet the home is the unit of society, and according to the strength, health and purity of the home will be the strength, health and purity of the nation. "By their fruits ye shall know them." Carefully read Judge Batts' scholarly paper in the last issue of the JOURNAL.

